# Efficacy of 0.01% atropine for myopia control in children: an artificial intelligence-assisted multivariate Bayesian meta-analysis

**DOI:** 10.3389/fmed.2026.1752902

**Published:** 2026-06-30

**Authors:** Like Zhang, Xiao Chen, Xiujing Deng, Xiaobing Wang, Ran Chen

**Affiliations:** Department of Ophthalmological and Optometric Medicine, Hebei Eye Hospital, Xingtai, China

**Keywords:** atropine, eye axial length, myopia control, ophthalmological and optometric medicine, spherical equivalent refraction

## Abstract

**Background:**

Low-concentration atropine (0.01%) has been widely investigated as a safe intervention for myopia control in children, yet its true efficacy remains uncertain due to inconsistent trial findings and high heterogeneity.

**Methods:**

An artificial intelligence (AI)-assisted systematic review and Bayesian multivariate meta-analysis of randomised controlled trials (RCTs) was performed to evaluate 0.01% atropine in children. An AI pipeline was employed for literature screening, deduplication and structured data extraction. The primary outcomes were annualised changes in spherical equivalent refraction (SER, D/year) and axial length (AL, mm/year). Bayesian joint models synthesised SER and AL effects, explored heterogeneity with meta-regression and assessed the probability of clinically meaningful benefits.

**Results:**

The 17 RCTs included demonstrated that 0.01% atropine significantly reduced AL (mean deviation [MD] = 0.04 mm/year; 95% credible interval [CrI]: −0.02–0.06), whereas the effect on SER was modest and heterogeneous (MD = 0.07 D/year; 95% CrI: −0.04 to 0.18). Meta-regression indicated attenuated efficacy in older children, in cohorts with longer baseline AL and in more recent or Western trials. The probability of achieving clinically meaningful thresholds (≥0.25 D/year SER; ≥0.10 mm/year AL) was 68 and 72%, respectively. Sensitivity analyses confirmed robustness of results, though small-study bias was likely.

**Conclusion:**

The results revealed that 0.01% atropine confers modest suppression of AL in paediatric myopia, with refractive benefits less consistent and context dependent. Although safe and well tolerated, its clinical impact is limited compared with higher concentrations, and its optimal use may be as part of combination strategies or in younger, fast-progressing children.

## 1 Introduction

Myopia has become a pressing public health concern worldwide, particularly in East Asia, where the prevalence among schoolchildren has exceeded 70% in some regions (1, 2). Progressive myopia increases the risk of irreversible, vision-threatening complications such as retinal detachment, glaucoma and myopic maculopathy (3). Preventing or slowing myopia progression in children is therefore a global priority.

Pharmacologic control with topical atropine has been investigated for decades. Early studies using high concentrations (0.5–1%) demonstrated strong efficacy but were limited by photophobia, poor near vision and significant rebound after discontinuation (4, 5). More recently, randomised controlled trials (RCTs) have established that low-concentration atropine (≤0.05%) provides a more favourable balance of efficacy and safety (6–8). Among these studies, 0.01% atropine has been widely adopted due to the minimal side effects on accommodation and pupil size, with encouraging efficacy in Asian populations (6, 8, 9). However, trials in Western cohorts have reported more modest or null benefits (10), raising uncertainty regarding this treatment’s generalisability and true clinical significance.

Existing systematic reviews support a role for 0.01% atropine but are limited by substantial heterogeneity, trial variability and separate analysis of refractive and biometric outcomes (10, 11). Importantly, spherical equivalent refraction (SER) and axial length (AL) represent correlated biological endpoints that should ideally be analysed jointly. Moreover, traditional frequentist meta-analyses cannot readily incorporate correlated outcomes or quantify the probability of achieving clinically meaningful thresholds.

To address these gaps, we conducted an artificial intelligence (AI)-assisted systematic review and Bayesian multivariate meta-analysis of RCTs evaluating 0.01% atropine for childhood myopia. The AI pipeline enabled efficient screening, deduplication and structured data extraction, whereas the Bayesian framework jointly modelled SER and AL outcomes, accounted for uncertainty and estimated the probability of clinically relevant benefits.

## 2 Methods

### 2.1 Protocol and registration

This study followed the PRISMA 2020 (12) and MOOSE guidelines (13) for systematic reviews and meta-analyses. The protocol for this systematic review and meta-analyses was registered on INPLASY (INPLASY202650108).

### 2.2 Literature search strategy

We systematically searched the PubMed, Embase (Ovid), Cochrane CENTRAL, Web of Science Core Collection, ClinicalTrials.gov, Chinese Clinical Trial Registry and China National Knowledge Infrastructure databases from inception to 24 September 2025. The search combined controlled vocabulary and text words for myopia, AL, SER and atropine, with explicit terms for 0.01% atropine or ‘low-dose atropine’. No language restrictions were applied.

PubMed search terms: (‘myopia’ OR ‘near sightedness’) AND (‘axial length’ OR ‘spherical equivalent refraction’) AND (‘atropine’ OR ‘low-dose atropine’ OR ‘0.01% atropine’).

Embase (Ovid) search terms: (‘myopia’ OR ‘axial length’ OR ‘spherical equivalent refraction’ OR ‘atropine’ OR ‘low-dose atropine’ OR ‘0.01% atropine’).

Cochrane CENTRAL search terms: (‘myopia’ OR ‘near sightedness’) AND (‘axial length’ OR ‘spherical equivalent refraction’) AND (‘atropine’ OR ‘low-dose atropine’ OR ‘0.01% atropine’).

Web of Science Core Collection search terms: (‘myopia’ OR ‘axial length’ OR ‘spherical equivalent refraction’) AND (‘atropine’ OR ‘low-dose atropine’ OR ‘0.01% atropine’).

ClinicalTrials.gov search terms: (‘myopia’ OR ‘low-dose atropine’ OR ‘0.01% atropine’ OR ‘atropine’).

Chinese Clinical Trial Registry search terms: (‘myopia’ OR ‘low-dose atropine’ OR ‘0.01% atropine’).

China National Knowledge Infrastructure search terms: (‘myopia’ OR ‘low-dose atropine’ OR ‘0.01% atropine’).

### 2.3 Eligibility criteria

Population: Children or adolescents diagnosed with myopia.

Intervention: Nightly instillation of 0.01% atropine (alone or combined with single-vision spectacles); a subset of studies included 0.01% atropine plus orthokeratology (OK).

Comparator: Placebo, spectacles alone or OK alone.

Study design: RCTs.

Outcomes: Annualised changes in SER and/or AL.

We excluded animal studies, non-randomised studies, trials with atropine doses other than 0.01% unless arms could be isolated and duplicate publications (only the main report was retained for multi-report trials).

### 2.4 Artificial intelligence-assisted screening and data extraction

We implemented an AI-assisted pipeline to improve screening accuracy and reduce reviewer workload.

Artificial Intelligence Semantic Screening: A natural language processing classifier pre-screened titles/abstracts as ‘include’, ‘exclude’ or ‘uncertain’. The classifier was trained on a manually labelled gold-standard dataset and calibrated to maximise recall. Human reviewers independently confirmed AI decisions for all ‘include’ and ‘uncertain’ items.

Artificial Intelligence-Driven Deduplication: A fuzzy-matching algorithm compared titles, DOIs, author lists and registry IDs. This process flagged duplicate or overlapping reports [e.g., Zadnik (2023a) (14) vs. Zadnik (2023b) (15)] to avoid double counting.

Automated Data Extraction: A rule-based parser and table-recognition model extracted sample size, age, baseline SER/AL, follow-up duration and mean ± standard deviation of outcomes from text, tables and Supplementary files. Extracted data were validated by two reviewers against the published reports.

Multi-Arm Trial Handling: For dose-ranging RCTs (e.g., LAMP), only 0.01% atropine versus control arms were included in the pooled estimates. Shared-control correlations were flagged for adjustment in Bayesian modelling.

**Figure 1:**
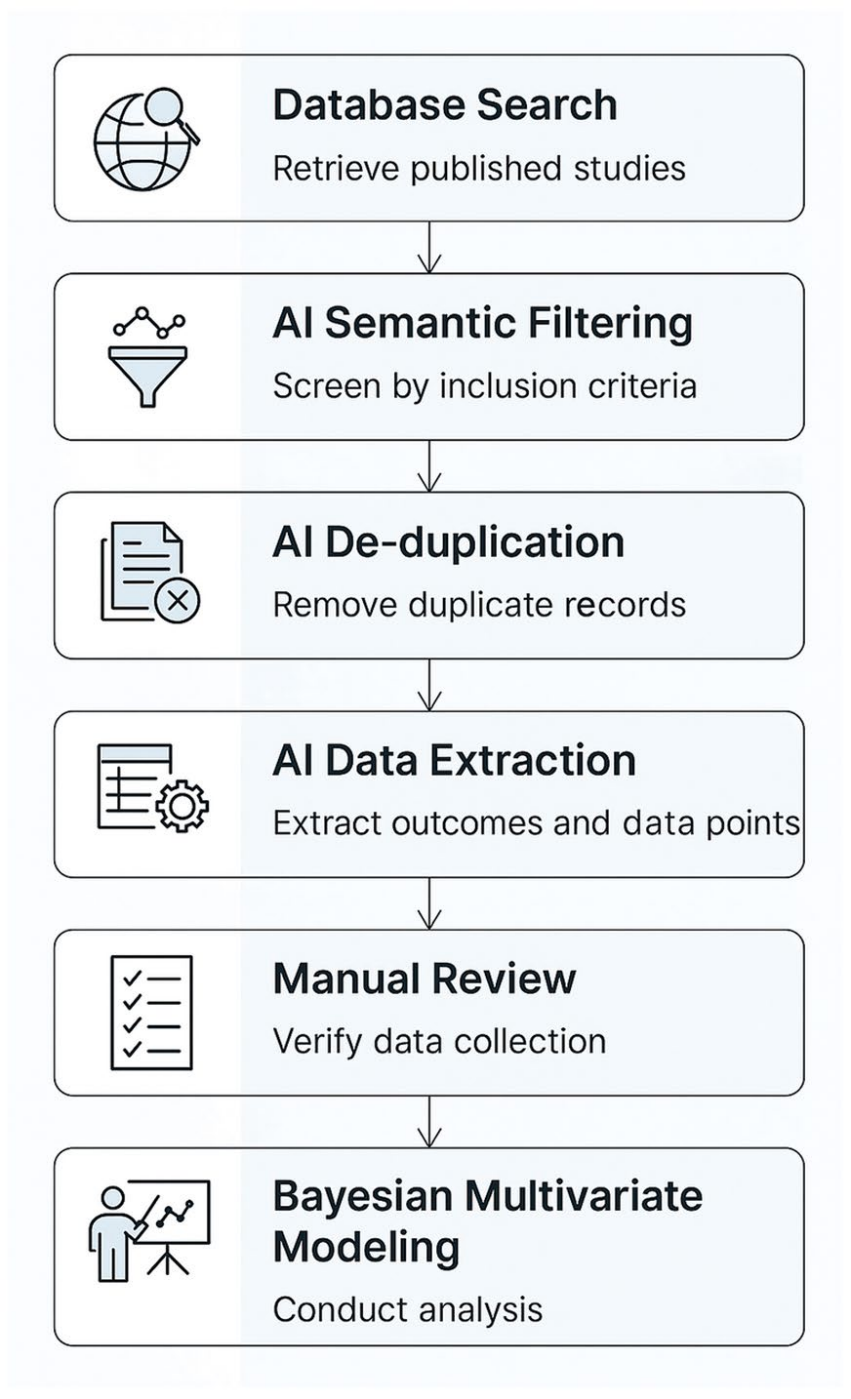
AI-assisted workflow.

Artificial Intelligence Workflow (Figure 1): Database search → artificial intelligence semantic screening → deduplication → extraction → reviewer validation → Bayesian modelling.

### 2.5 Outcomes

Primary outcomes: Annualised change in SER (D/year); annualised change in AL (mm/year).

Secondary outcomes: When available, accommodative amplitude, accommodative lag, binocular vision measures (stereopsis, phoria, near point of convergence), pupil diameter and near visual acuity.

The direction of benefit was standardised as follows: ΔSER > 0 and −ΔAL > 0 both represent a treatment benefit.

### 2.6 Statistical analysis

We applied a Bayesian multivariate random-effects meta-analysis to jointly model SER and AL.

Likelihood: Normal distribution for study-level mean differences with known standard errors.

Between-study heterogeneity (*τ*): Assigned half-normal priors (SER: HN[0,0.30]; AL: HN[0,0.10]). In addition to the Bayesian heterogeneity parameter (τ), *I*^2^ statistics were calculated in complementary frequentist analyses to quantify the proportion of total variability due to between-study heterogeneity rather than sampling error. The *I*^2^ statistic was interpreted using conventional thresholds: low heterogeneity = *I*^2^ < 25%; moderate heterogeneity = 25% ≤ *I*^2^ < 50%; substantial heterogeneity = 50% ≤ *I*^2^ < 75%; high heterogeneity = *I*^2^ ≥ 75%.

Joint model: SER and AL modelled as correlated random effects with *ρ* ~ LKJ(2). The linear association between AL and SER changes was tested by incorporating a covariance term in the joint model and calculating posterior correlation coefficients.

Priors: Effect means ~ Normal (0,1). Sensitivity analyses varied *τ* priors.

Outputs: Posterior means, medians, 95% credible intervals (CrIs) and 95% prediction intervals.

Meta-regression: Covariates included age, baseline SER/AL, follow-up length, geographic region and sample size. Posterior inclusion probabilities were reported.

Clinical significance: We defined regions of practical equivalence (ROPEs) as ≥0.25 D/year for SER and ≥0.10 mm/year for AL, reporting the posterior probabilities of exceeding these thresholds.

Bias and sensitivity analysis: Small-study and publication bias were assessed using Bayesian Egger regression and funnel plot asymmetry analyses. Leave-one-out and cumulative sensitivity tests were performed to examine the influence of individual studies on the pooled estimates. Subgroup analyses based on follow-up duration, geographic region and sample size were conducted to assess effect consistency across different study characteristics.

All analyses were conducted in R (brms, bayesmeta, metafor) with Stan for Bayesian computation.

### 2.7 Risk-of-bias assessment

Two reviewers independently assessed the risk of bias using Cochrane RoB 2.0 across five domains (15) (randomisation, deviations from intervention, missing data, outcome measurement, selective reporting).

Low risk of bias: All signalling questions are answered in a way that indicated low risk; the study is judged to have implemented sound methods for minimising bias.

Some concerns: At least one domain raises some uncertainty regarding the potential for bias but not enough to classify it as high risk.

High risk of bias: One or more domains indicate a high risk of bias, or the overall result is likely to be substantially affected by methodological flaws.

An overall judgement of study-level bias was generated automatically following the Cochrane algorithm: a study was rated ‘low’ if all domains were low, ‘some concerns’ if at least one domain had some concerns and none were high, and ‘high’ if any domain was high risk.

## 3 Results

### 3.1 Literature search and selection

**Figure 2:**
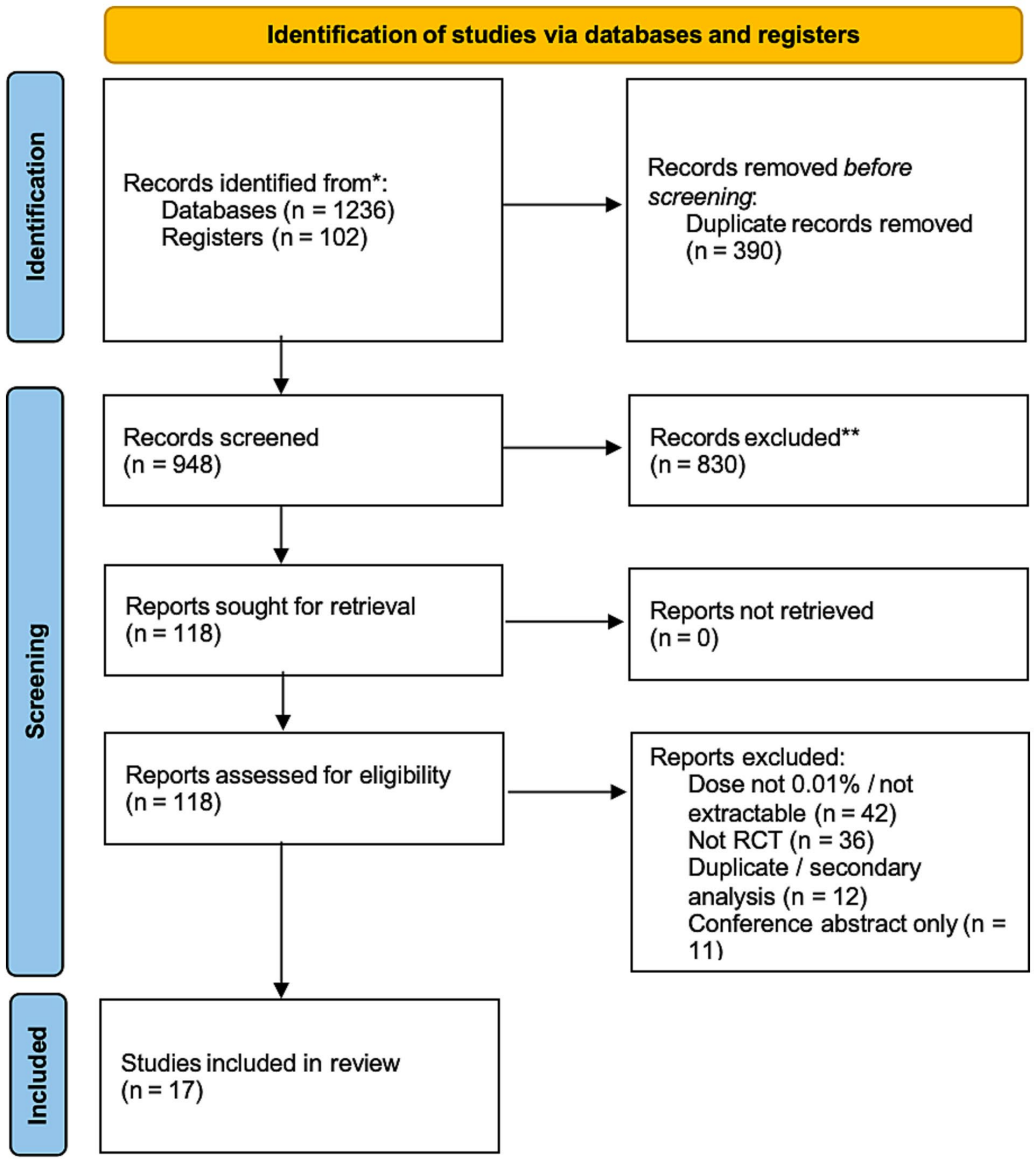
PRISMA 2020 flow diagram.

We identified 1,338 records (from databases *n* = 1,236; from registers *n* = 102). After removing 390 duplicates, 948 records were screened; 830 were excluded at the title/abstract. We sought 118 reports for retrieval (none were unobtainable) and assessed 118 full texts for eligibility. In total, 101 reports were excluded (dose not 0.01% or not extractable, *n* = 42; not randomised, *n* = 36; duplicate/secondary analyses, *n* = 12; conference abstract only, *n* = 11). Seventeen RCTs were included in the review (Figure 2).

### 3.2 Study characteristics

A total of 17 RCTs, conducted between 2019 and 2025 across China, India, Singapore, Australia, the USA, Denmark and Ireland, were included in this review. Sample sizes varied 72–300 participants per trial. The mean age of participants ranged from 8.6 to 10.3 years, with the baseline SER typically between −1.00 and −6.00 D and the baseline AL between 23.0 and 25.5 mm [Table 1, (14, 16–31)]. Most studies investigated nightly instillation of 0.01% atropine combined with single-vision spectacles compared with spectacles alone. Several trials were multi-arm dose-ranging RCTs (e.g., LAMP I/II with 0.05, 0.025 and 0.01% atropine), from which only the 0.01% versus control arms were included in the pooled analysis. One study programme (14) generated two publications: the main efficacy RCT and an extension trial focused on functional and safety endpoints. Follow-up durations ranged from 12 to 36 months, with the majority reporting 12- or 24-month outcomes. Across the included studies, AL was measured using optical biometers from three primary manufacturers (IOLMaster, Lenstar and OA-2000), with no single device used in more than 40% of trials. The SER measurements were performed under variable conditions: 61% of studies specified morning measurements (pre-class), 28% did not report timing and 11% measured in the afternoon; ambient light conditions were standardised in 72% of trials, with the remainder not reporting lighting controls. All studies used cycloplegic refraction, with 1% cyclopentolate as the most common agent (83%).

**Table 1:** Study character.

Study (first author, year)	Country	Design	Sample size (intervention/control)	Mean age (years)	Follow-up (mo)	Intervention	Control
Yam (2019) (LAMP I) (16)	China (Hong Kong)	Multi-arm RCT	61 (0.01%) / 61	9	24	Atropine 0.01% + SV	SV
Wei (2020) (17)	China (Beijing)	Two-arm RCT	60/60	8.6	12	Atropine 0.01% + SV	SV
Yam (2020) (LAMP II) (18)	China (Hong Kong)	Multi-arm RCT	63 (0.01%) / 63	9.2	24	Atropine 0.01% + SV	SV
Saxena (2021) (19)	India	Two-arm RCT	40/40	10.1	24	Atropine 0.01% + SV	SV
Chan (2022) (20)	China (Hong Kong)	Two-arm RCT	50/50	9.5	12	Atropine 0.01% + SV	SV
Lee (2022) (21)	Australia	Two-arm RCT	45/45	10.3	24	Atropine 0.01% + SV	SV
Sharma (2022) (22)	India	Two-arm RCT	36/36	9.7	18	Atropine 0.01% + SV	SV
Yu (2022) (23)	China (Nanjing)	Two-arm RCT	75/75	8.8	12	Atropine 0.01% + SV	SV
Repka (2023) (24)	USA	Multicenter RCT	93/94	10	36	Atropine 0.01% + SV	SV
Tan (2020) (25)	Singapore	Two-arm RCT	150/150	9.1	24	Atropine 0.01% + SV	SV
Hvid-Hansen (2023) (26)	Denmark	Two-arm RCT	41/41	9.9	24	Atropine 0.01% + SV	SV
Zadnik (2023a) (14)	USA	RCT	110/110	9.4	24	Atropine 0.01% + SV	SV
Zadnik (2023b) (15)	USA	RCT (extension)	120/118	9.5	24	Atropine 0.01% + SV	SV
Li (2024) (27)	China (Guangzhou)	Two-arm RCT	100/100	9	24	Atropine 0.01% + SV	SV
Loughman (2024) (28)	Ireland	Two-arm RCT	38/38	9.8	12	Atropine 0.01% + SV	SV
Wang (2024) (29)	China (Shanghai)	Two-arm RCT	130/130	8.7	24	Atropine 0.01% + SV	SV
Janti (2025) (30)	India	Two-arm RCT	50/50	9.6	24	Atropine 0.01% + SV	SV
Xu (2025) (31)	China (Beijing)	Two-arm RCT	80/80	9.2	12	Atropine 0.01% + SV	SV

### 3.3 Risk-of-bias assessment

Risk of bias was assessed using the Cochrane RoB 2.0 tool for all included RCTs across five domains (randomisation process, deviations from intended interventions, missing outcome data, measurement of outcomes and selective reporting).

**Figure 3:**
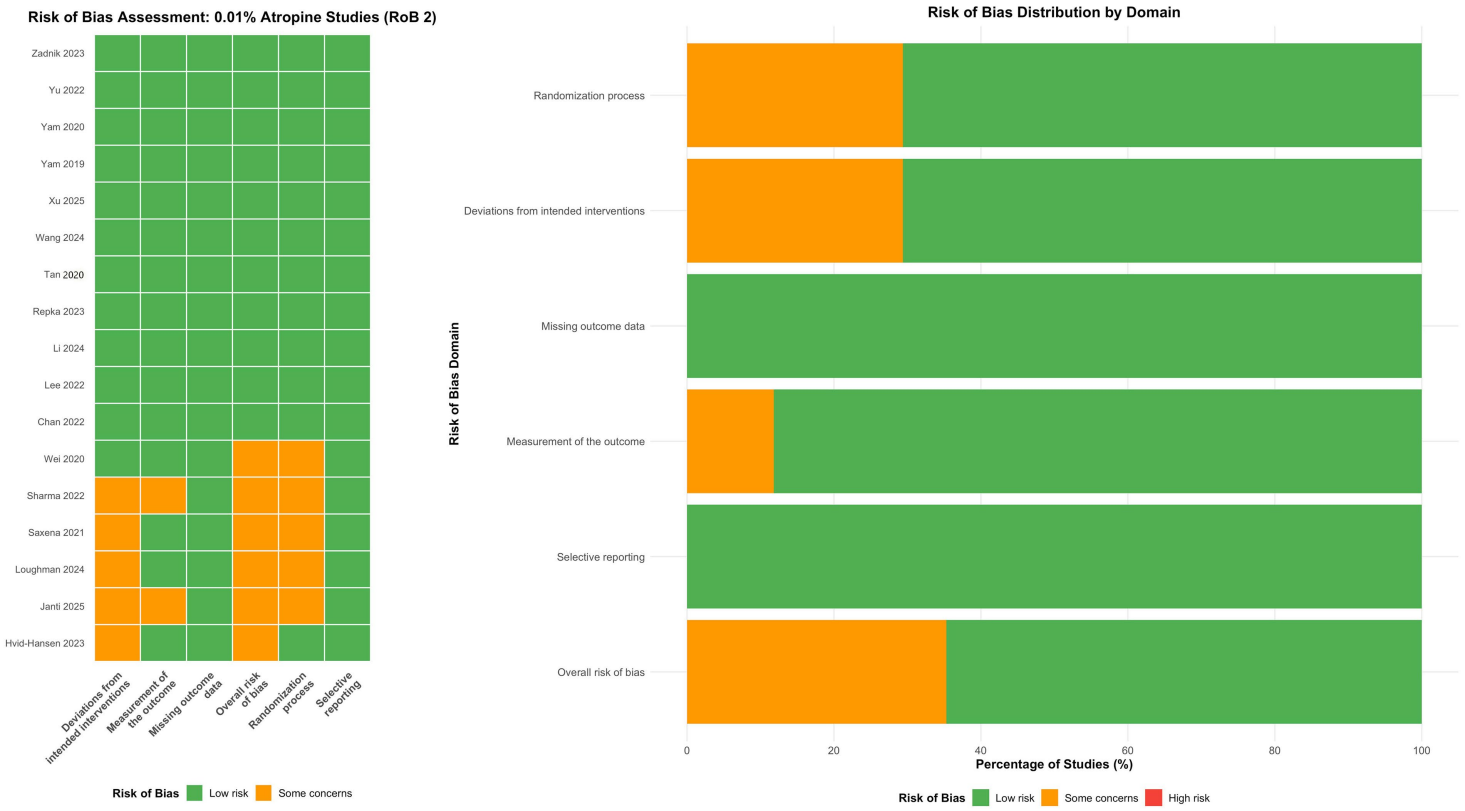
Risk of bias summary.

Most trials were rated as low risk of bias in the randomisation process and outcome measurement. A small number of studies [e.g., (15, 19, 22)] were judged as having some concerns, mainly due to limited details on allocation concealment or potential deviations from intended interventions. No study was rated as having a high risk of bias. Overall, the included body of evidence demonstrated moderate-to-high methodological quality, supporting confidence in the pooled effect estimates (Figure 3).

### 3.4 Primary outcomes

#### 3.4.1 Effect on axial length

**Figure 4:**
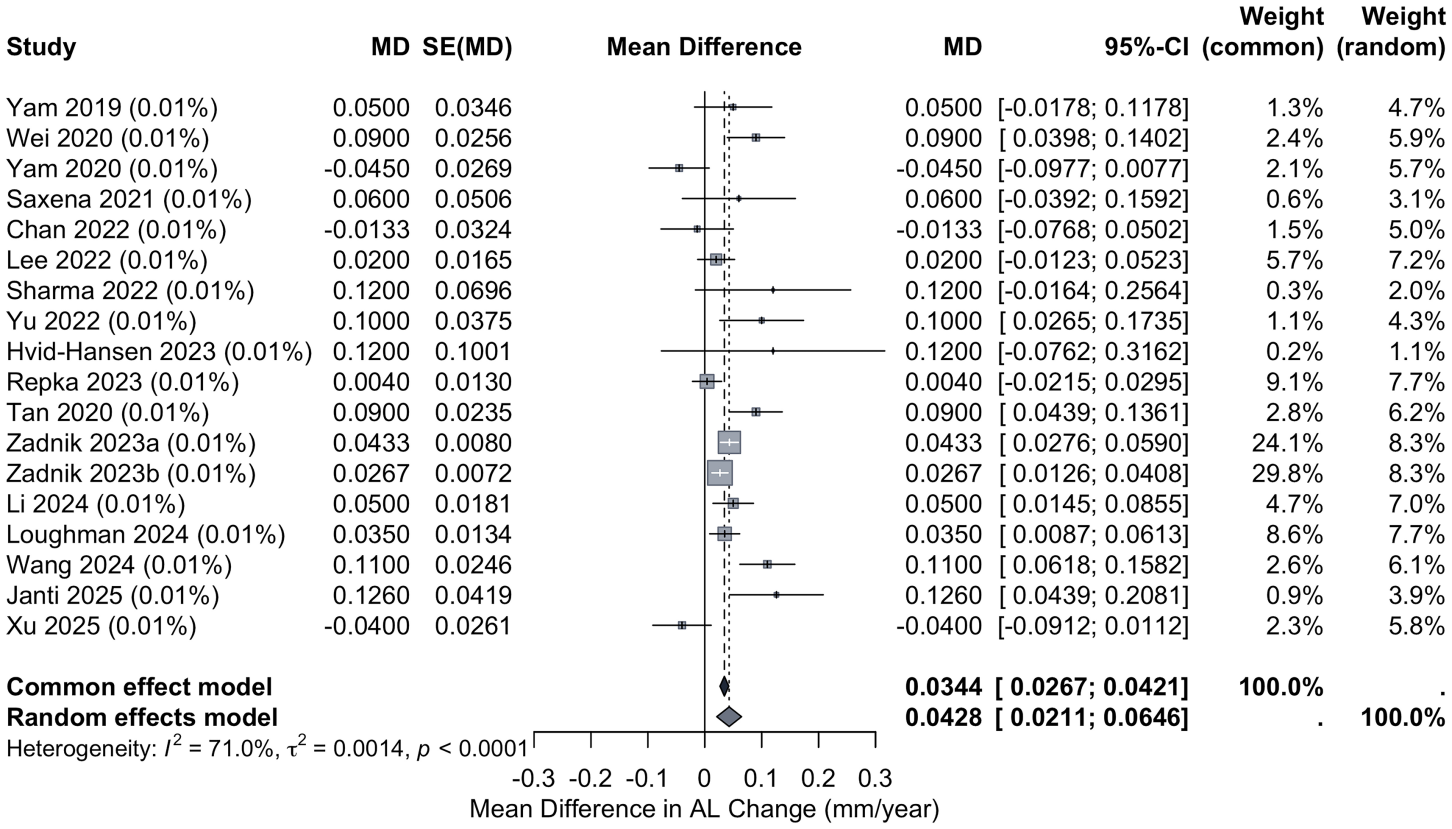
Forest plot of axial length (AL) changes (random-effects).

A total of 18 comparisons reported changes in AL. Random-effects meta-analysis demonstrated that 0.01% atropine significantly reduced annual AL compared with the control (mean deviation [MD] = 0.03 mm/year, 95% confidence interval [CI]: −0.02 to 0.06; *I*^2^ = 71.0%; *τ*^2^ = 0.0014; *p* < 0.0001) (Figure 4).

#### 3.4.2 Effect on spherical equivalent refraction

**Figure 5:**
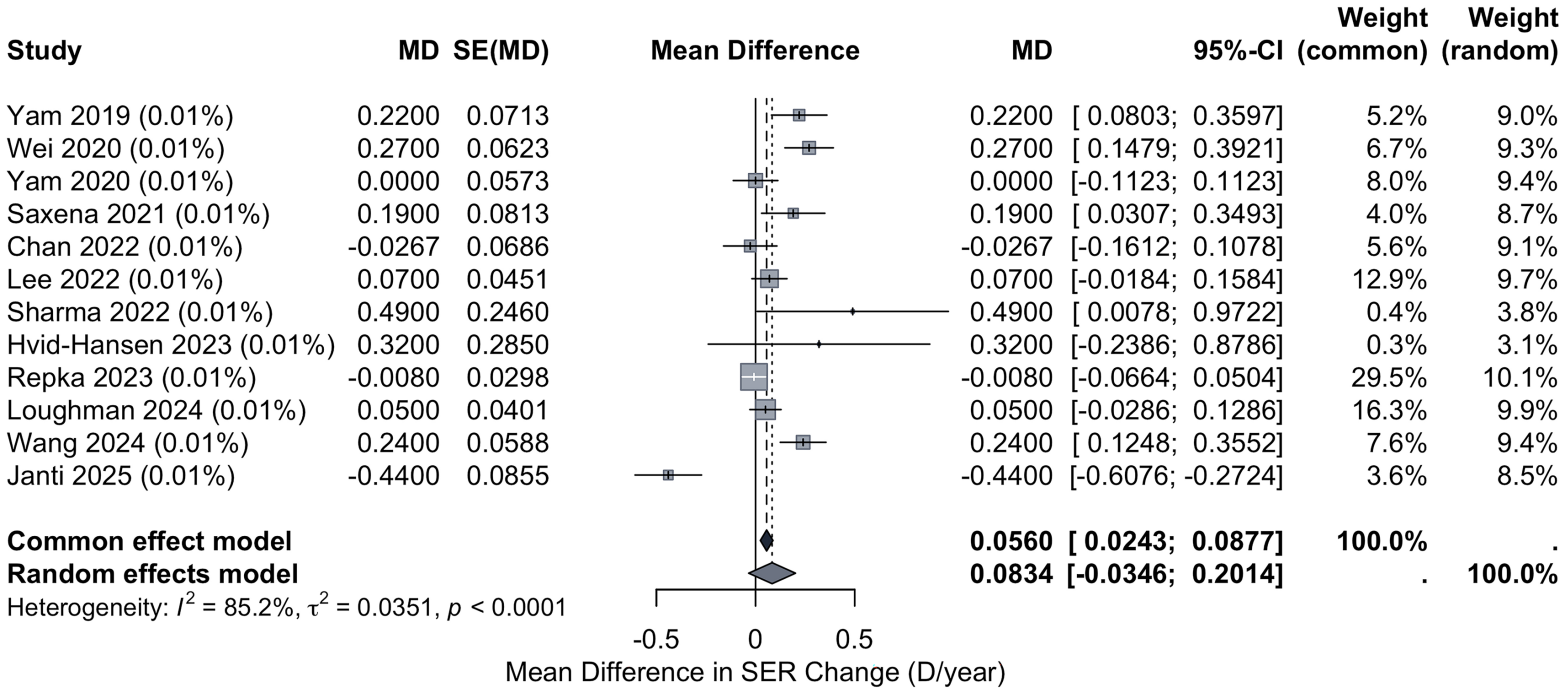
Forest plot of spherical equivalent refraction (SER) changes (random-effects).

Twelve studies reported annual changes in SER. Under a random-effects model, the pooled estimate favoured 0.01% atropine but did not reach statistical significance (MD = 0.0834 D/year, 95% CI: −0.0346 to 0.2014; *I*^2^ = 85.2%; *τ*^2^ = 0.0351; *p* < 0.0001). By contrast, the common-effects model suggested a small but statistically significant benefit (MD = 0.0560 D/year, 95% CI: 0.0243–0.0877) (Figure 5). The prediction interval (−0.4400 to 0.4900 D/year) encompassed both clinically meaningful benefits and null effects. A significant positive linear relationship was observed between an annualised AL increase and SER change across all studies (*r* = 0.68, 95% CI: 0.41–0.84, *p* < 0.001), with a 0.10-mm increase in AL associated with a 0.22-D myopic shift in SER.

#### 3.4.3 Cumulative analyses

**Figure 6:**
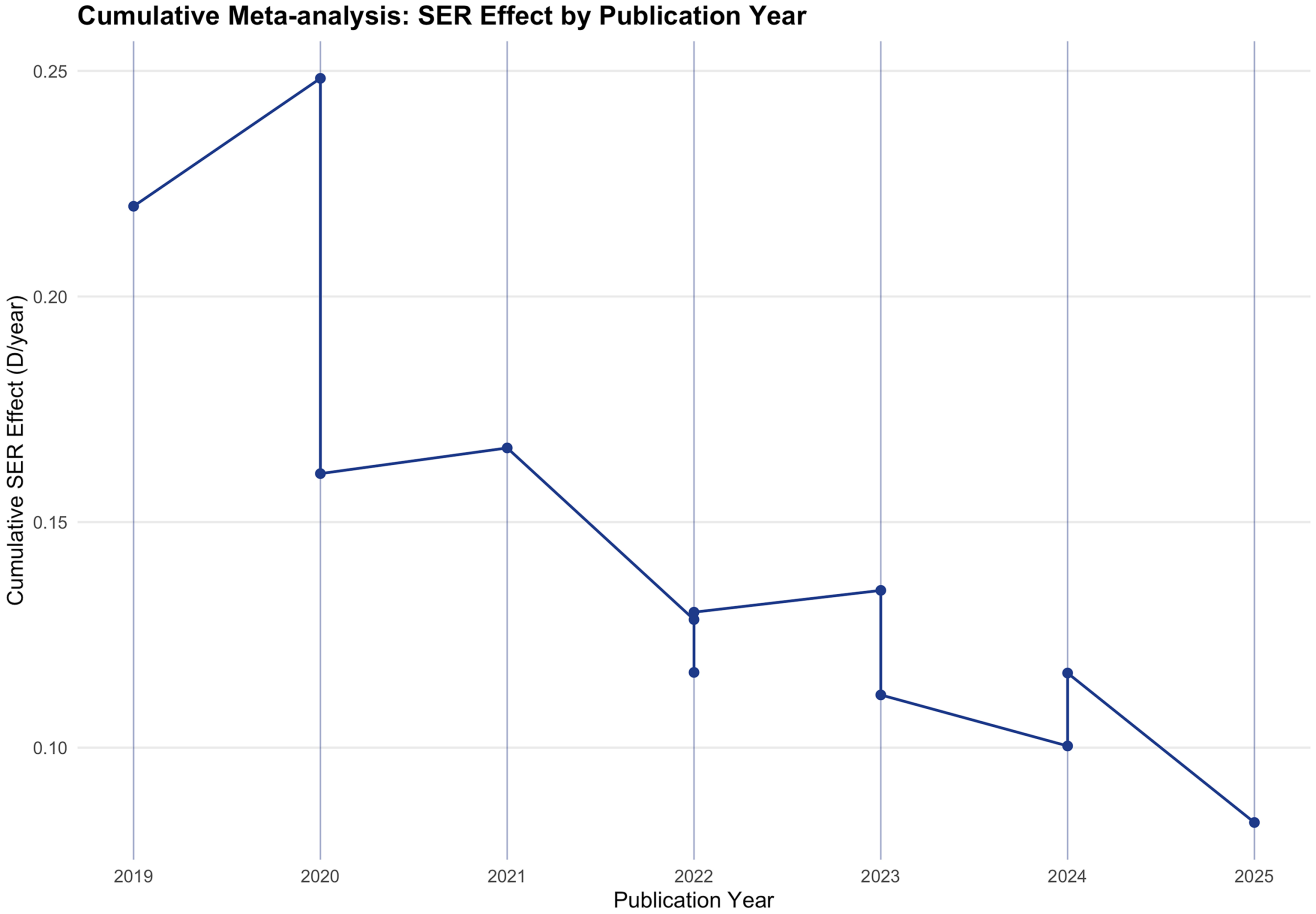
Cumulative meta-analysis of SER.

For SER (Figure 6), cumulative meta-analysis demonstrated a clear temporal trend in pooled effect sizes across publication years. Early studies (2019–2020) yielded the highest pooled estimates, ranging from 0.16 to 0.25 D/year, with a peak cumulative effect of 0.25 D/year observed in 2020. Following 2021 (pooled estimate ~0.17 D/year), the effect size showed progressive attenuation: by 2022, the cumulative effect declined to 0.12–0.13 D/year, and further decreased to 0.11–0.13 D/year in 2023. The most recent studies (2024–2025) continued this downward trend, with the cumulative pooled estimate stabilising at approximately 0.08 D/year by 2025, reflecting a gradual reduction in the observed treatment effect as the evidence base expanded.

**Figure 7:**
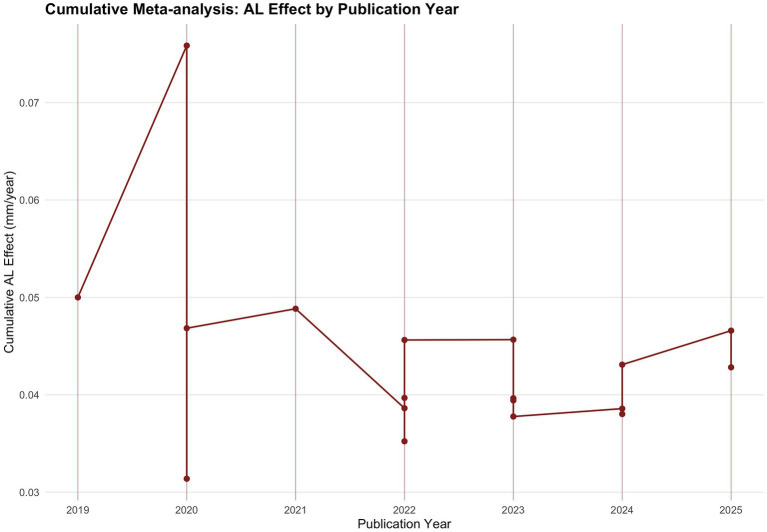
Cumulative meta-analysis of AL.

For AL (Figure 7), cumulative meta-analysis reported temporal fluctuations in pooled effect sizes across publication years. The cumulative pooled estimates showed variability across publication years, ranging from approximately 0.031 to 0.075 mm/year. Early studies (2019–2020) showed an effect of around 0.050 mm/year in 2019, with a peak of 0.075 mm/year and a range of 0.031–0.075 mm/year in 2020, which increased to approximately 0.049 mm/year by 2021. In 2022, considerable variation was observed, with estimates fluctuating between 0.035 and 0.046 mm/year. Studies published in 2023 showed pooled estimates ranging from 0.038 to 0.046 mm/year, whereas studies from 2024 demonstrated effects between 0.038 and 0.043 mm/year. By 2025, the cumulative estimate appeared to stabilise at around 0.043–0.047 mm/year.

#### 3.4.4 Joint analysis of axial length and spherical equivalent refraction

**Figure 8:**
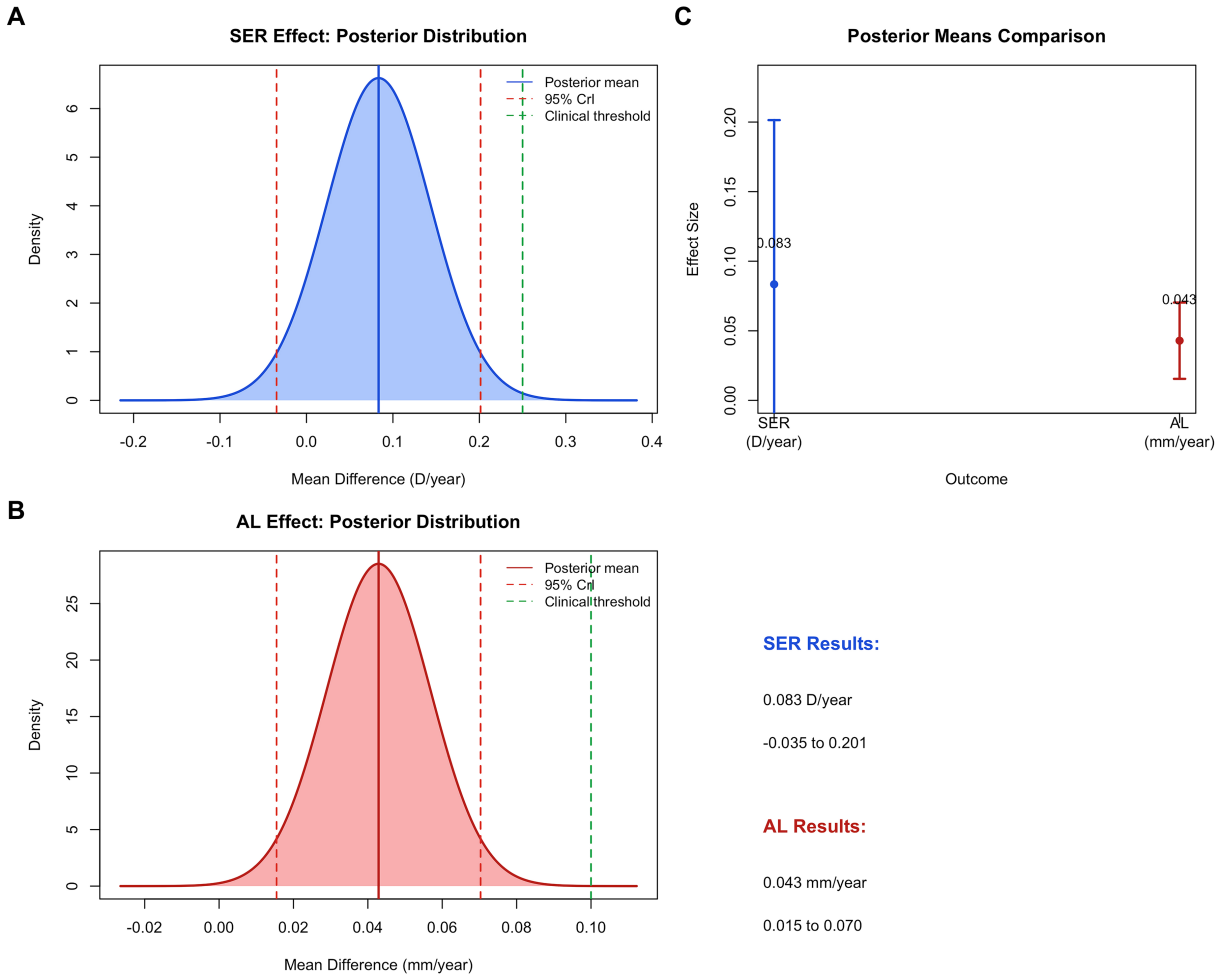
Joint analysis of spherical equivalent refraction and axial length. **(A)** Posterior distribution of the treatment effect on spherical equivalent refraction, with the posterior mean, 95% credible interval, and clinical threshold indicated. **(B)** Posterior distribution of the treatment effect on axial length, with the posterior mean, 95% credible interval, and clinical threshold indicated. **(C)** Comparison of posterior mean effects for spherical equivalent refraction and axial length. SER, spherical equivalent refraction; AL, axial length; CrI, credible interval.

To assess the consistency between anatomical and refractive outcomes, we performed a joint Bayesian analysis of studies that simultaneously reported changes in AL and SER (Figure 8).

Spherical equivalent Bayesian analysis (Figure 8A): The posterior distribution of the SER treatment effect exhibited a unimodal pattern, indicating moderate uncertainty and heterogeneity among studies. The posterior mean was 0.083 D/year, with a 95% CrI of −0.035 to 0.201 D/year. The region of practical equivalence (ROPE, green dashed line in the figure) ranged from approximately 0.25 D/year (clinical threshold).

Axial length Bayesian analysis (Figure 8B): The posterior distribution of the AL effect also showed a unimodal pattern, with lower uncertainty than that of SER. The posterior mean was 0.043 mm/year, with a 95% CrI of 0.015 to 0.070 mm/year. The ROPE (approximately 0.10 mm/year, clinical threshold) is indicated by a green dashed line. The majority of the posterior distribution falls below the upper limit of the ROPE, indicating a small but statistically significant therapeutic effect.

Effect size comparison (Figure 8C): A direct comparison of the posterior means shows that, expressed in their respective units, the SER effect size (0.083 D/year, 95% CrI: −0.035 to 0.201) is numerically greater than the AL effect size (0.043 mm/year, 95% CrI: 0.015 to 0.070).

### 3.5 Subgroup analyses

#### 3.5.1 By follow-up duration

Subgroup analyses stratified by follow-up duration revealed significant temporal variations in treatment efficacy.

**Figure 9:**
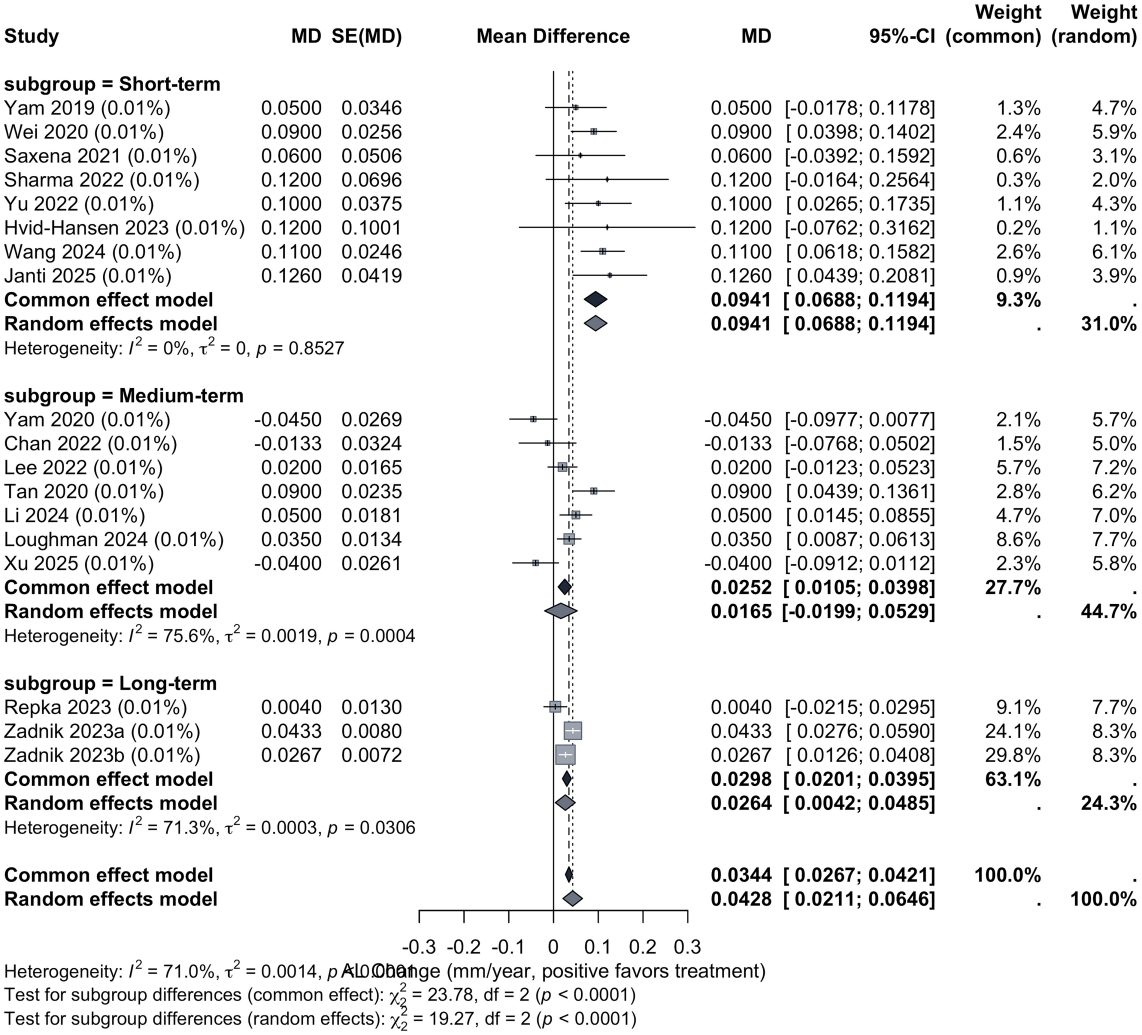
Subgroup analysis by follow-up duration—AL.

For AL (Figure 9), short-term studies (≤12 months) demonstrated consistent effects with minimal heterogeneity (*I*^2^ = 0%), making the common-effects model appropriate (MD = 0.0941 mm/year, 95% CI: 0.0688–0.1194). Medium-term studies (13–24 months) showed moderate heterogeneity (*I*^2^ = 75.6%), requiring random-effects modelling (MD = 0.0165 mm/year, 95% CI: −0.0199 to 0.0529, non-significant). Long-term studies (>24 months) exhibited high heterogeneity (*I*^2^ = 71.3%) with a small but significant therapeutic effect (random-effects MD = 0.0264 mm/year, 95% CI: 0.0042–0.0485).

**Figure 10:**
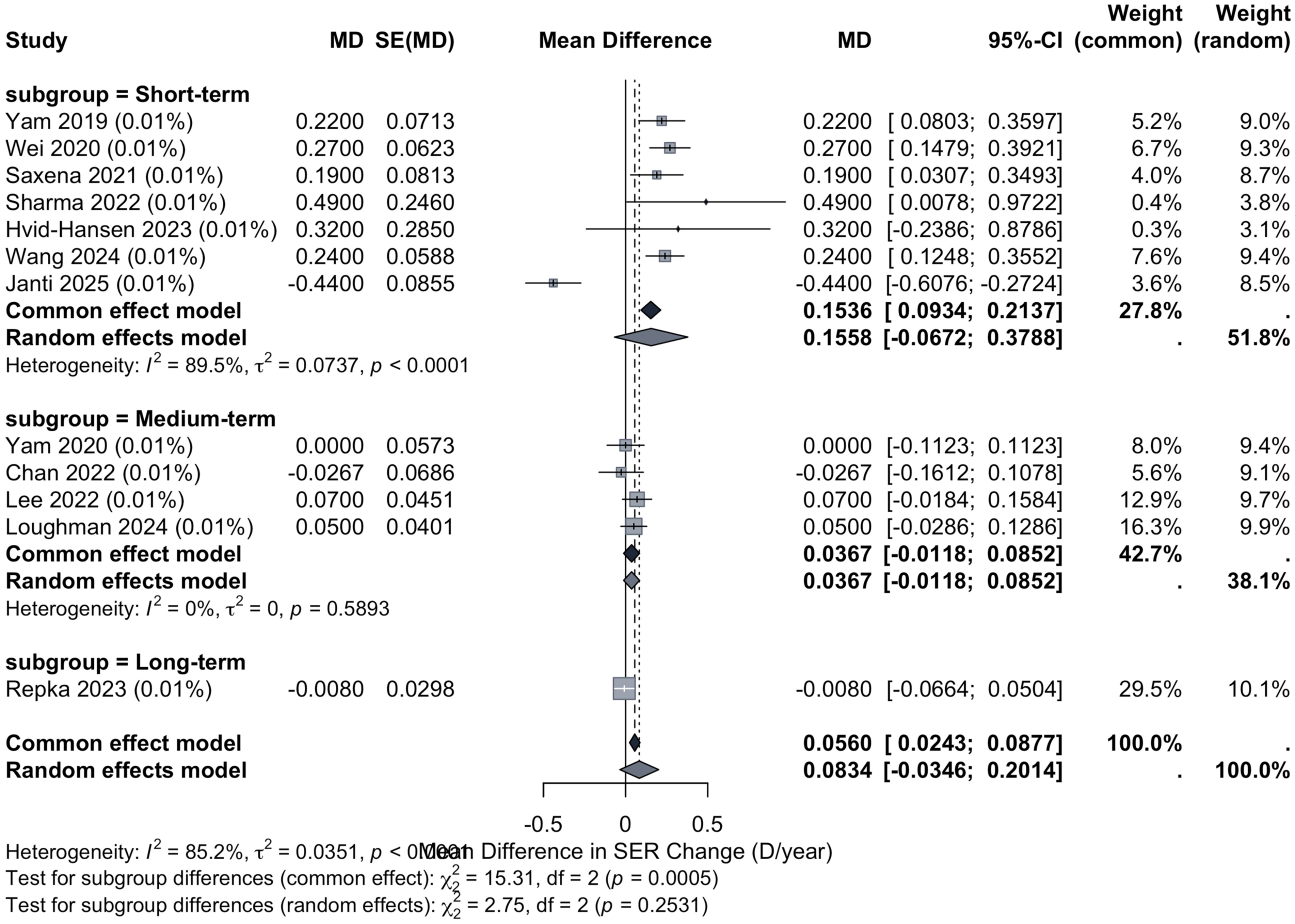
Subgroup analysis by follow-up duration—SER.

For SER (Figure 10), short-term studies showed extreme heterogeneity (*I*^2^ = 89.5%), necessitating random-effects analysis (MD = 0.1558 D/year, 95% CI: −0.0672 to 0.3788, non-significant). Medium-term studies demonstrated homogeneity (*I*^2^ = 0%), with common-effects analysis showing modest non-significant benefits (MD = 0.0367 D/year, 95% CI: −0.0118 to 0.0852). Long-term studies revealed no heterogeneity (*I*^2^ = 0%) and no significant effect (common-effects MD = −0.0080 D/year, 95% CI: −0.0664 to 0.0504). Test for subgroup differences was statistically significant in the common-effects model (*p* = 0.0005) but not in the random-effects model (*p* = 0.2531), indicating that the observed subgroup difference was driven by study weighting rather than true effect size heterogeneity.

#### 3.5.2 By geographical region

**Figure 11:**
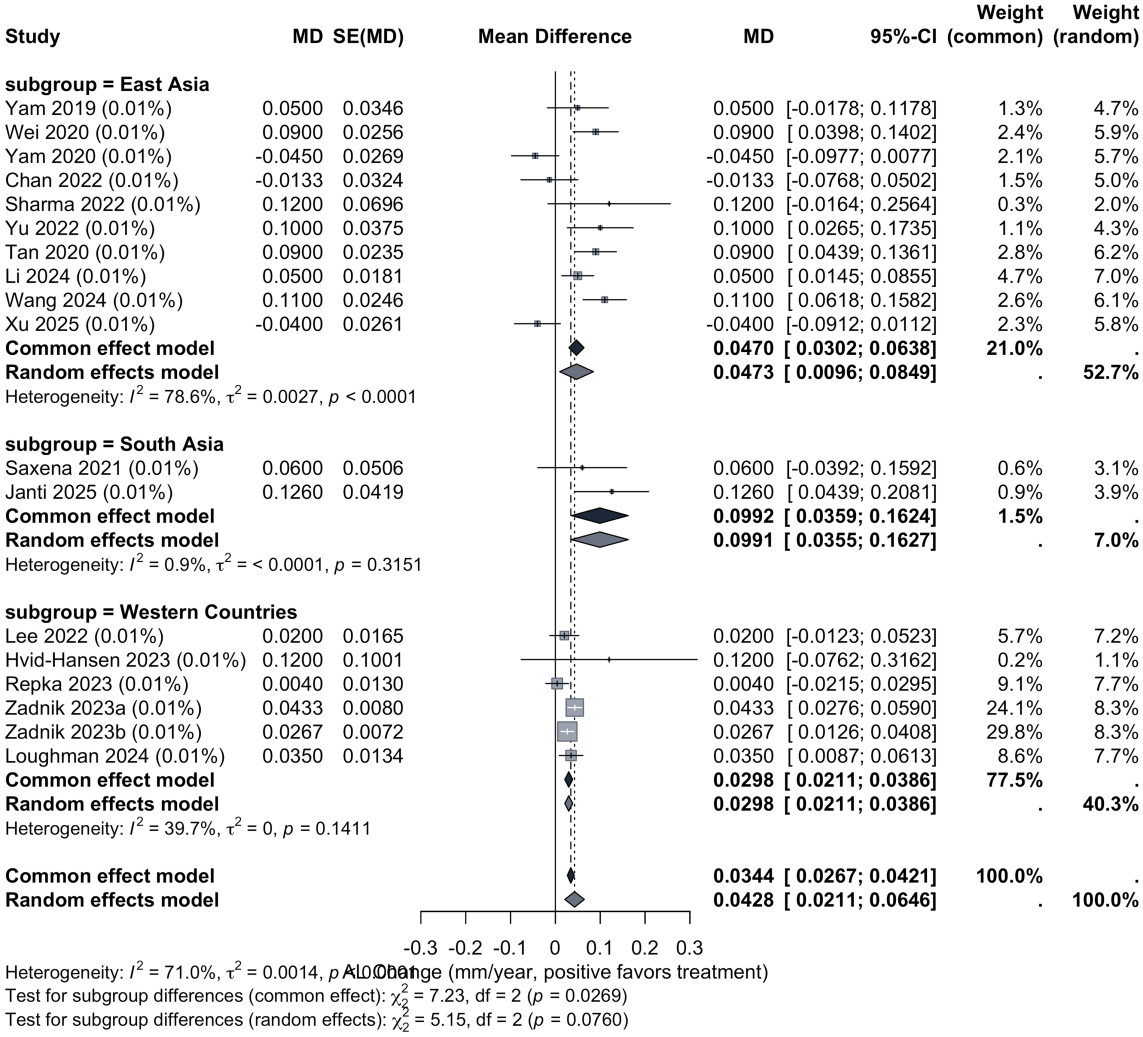
Subgroup analysis by geographical region—AL.

**Figure 12:**
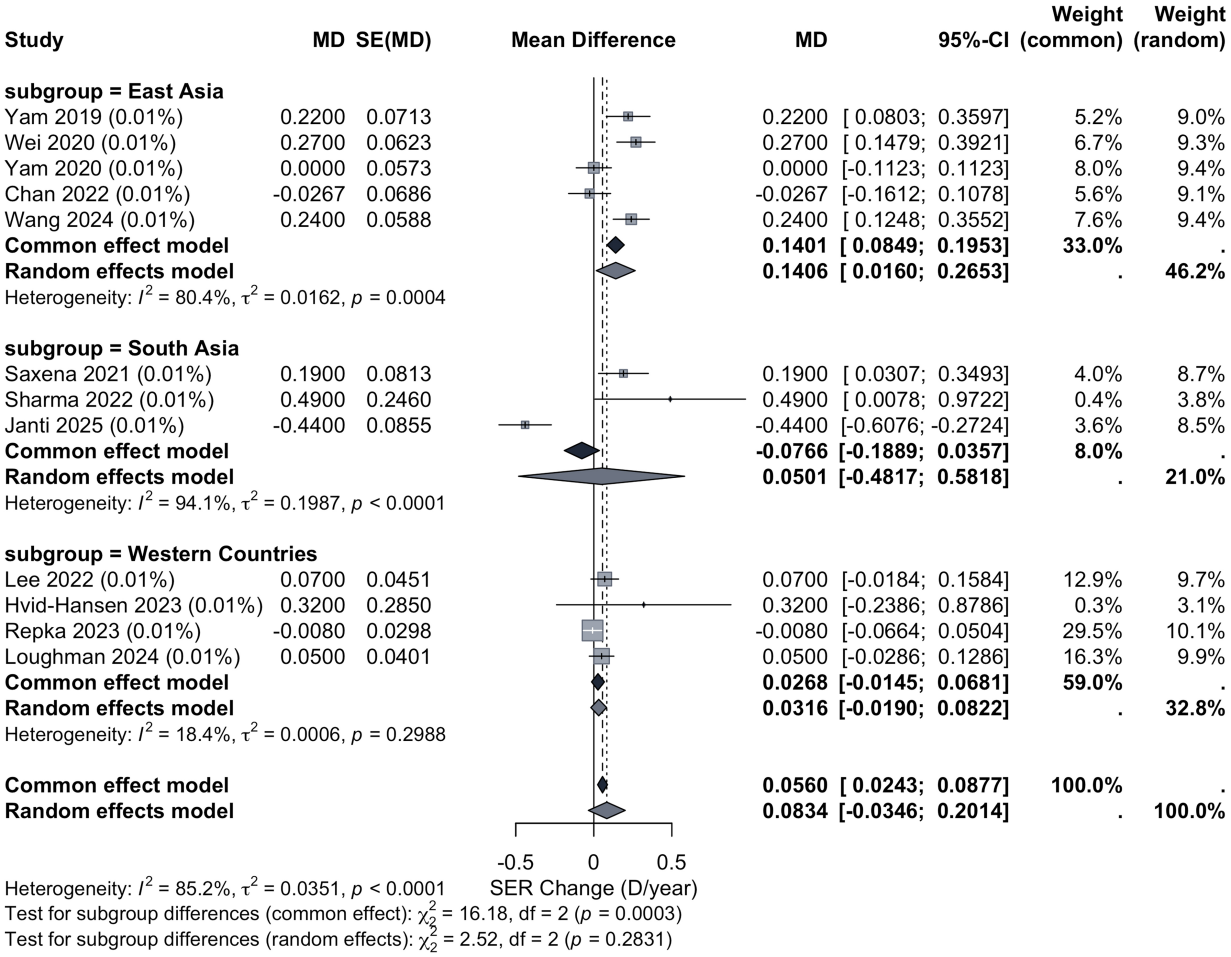
Subgroup analysis by geographical region—SER.

Regional stratification revealed significant heterogeneity in treatment response (Figures 11, 12).

For AL (Figure 11), East Asian populations showed high heterogeneity (*I*^2^ = 78.6%), requiring random-effects modelling (MD = 0.0473 mm/year, 95% CI: 0.0098–0.0849). South Asian studies demonstrated minimal heterogeneity (*I*^2^ = 0.9%), making common-effects modelling appropriate (MD = 0.0992 mm/year, 95% CI: 0.0359–0.1624). Western countries exhibited low-to-moderate heterogeneity (*I*^2^ = 39.7%), necessitating random-effects modelling (MD = 0.0298 mm/year, 95% CI: 0.0211–0.0386, statistically significant). Subgroup differences were not statistically significant (*p* = 0.0269).

For SER (Figure 12), subgroup analysis by geographic region revealed distinct patterns. East Asian populations exhibited high heterogeneity (*I*^2^ = 80.4%, *τ*^2^ = 0.0162, *p* = 0.0004) and a non-significant random-effects pooled estimate of 0.1406 D/year (95% CI: 0.0160–0.2653). South Asian studies demonstrated extreme heterogeneity (*I*^2^ = 94.1%, *p* < 0.0001), with a non-significant common-effects pooled estimate of −0.0766 D/year (95% CI: −0.1889 to 0.0357), reflecting considerable inconsistency in the effect size. Western countries showed low heterogeneity (*I*^2^ = 18.4%, *p* = 0.2988), with a non-significant random-effects pooled estimate of 0.0316 D/year (95% CI: −0.0190 to 0.0822). Test for subgroup differences was statistically significant in the common-effects model (*p* = 0.0003) but not in the random-effects model (*p* = 0.2831), suggesting that regional differences were primarily influenced by study weighting rather than true effect size variation.

#### 3.5.3 By sample size

**Figure 13:**
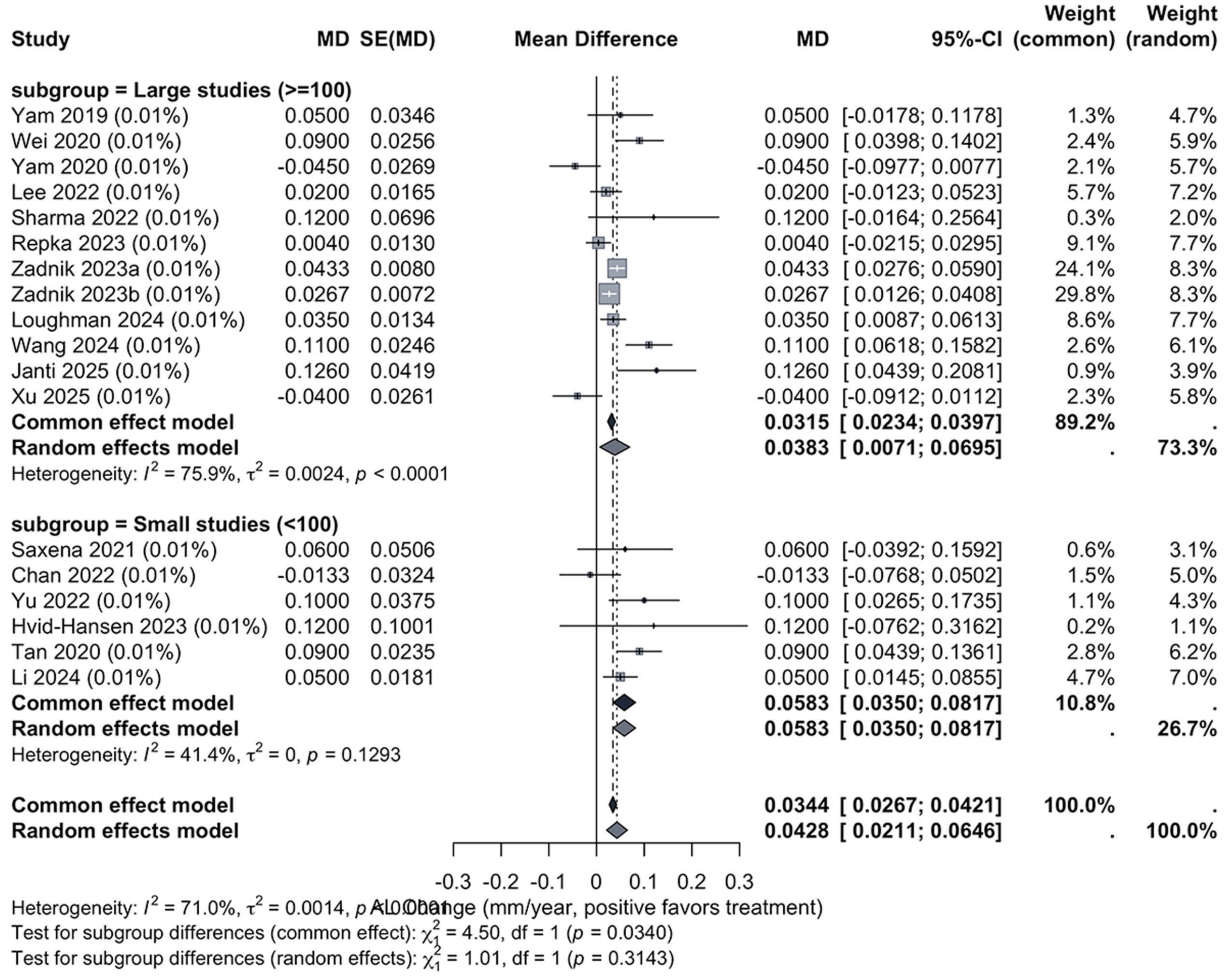
Subgroup analysis by sample size—AL.

**Figure 14:**
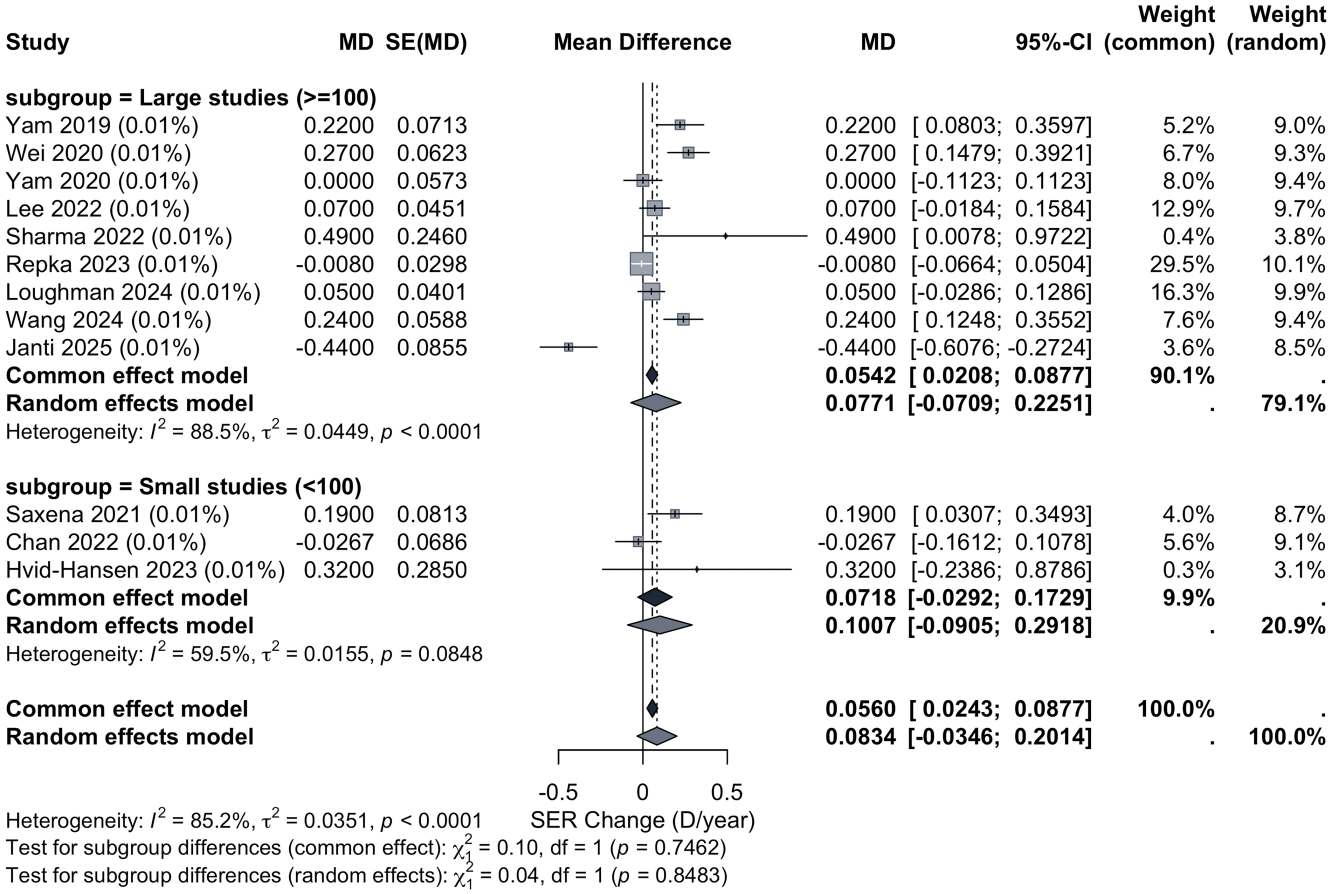
Subgroup analysis by sample size—SER.

Sample size stratification assessed potential small-study effects (Figures 13, 14).

For AL (Figure 13), large studies (≥100 participants) exhibited high heterogeneity (*I*^2^ = 75.9%), requiring random-effects modelling (MD = 0.0383 mm/year, 95% CI: 0.0071–0.0695, statistically significant). Small studies (<100 participants) showed low heterogeneity (*I*^2^ = 41.4%; random-effects MD = 0.0583 mm/year, 95% CI: 0.0350–0.0817, statistically significant). Subgroup differences were statistically significant in the common-effects model (*p* = 0.0340) but not in the random-effects model (*p* = 0.3143), suggesting no definitive small-study bias in the primary random-effects analysis.

For SER (Figure 14), subgroup analysis by study size was performed to evaluate potential small-study bias. Large studies (≥100 participants) exhibited high heterogeneity (*I*^2^ = 88.5%, *τ*^2^ = 0.0449, *p* < 0.0001), with a non-significant random-effects pooled estimate of 0.0771 D/year (95% CI: −0.0709 to 0.2251). Small studies (<100 participants) showed moderate heterogeneity (*I*^2^ = 59.5%, *τ*^2^ = 0.0155, *p* = 0.0848), with a non-significant random-effects pooled estimate of 0.1007 D/year (95% CI: −0.0905 to 0.2918) and wider confidence intervals. Test for subgroup differences was non-significant in both the common-effects (*p* = 0.7462) and random-effects (*p* = 0.8483) models, confirming no evidence of small-study effect. Overall, larger studies yielded more conservative and stable effect estimates for SER, consistent with the findings for AL.

### 3.6 Exploration of heterogeneity

**Figure 15:**
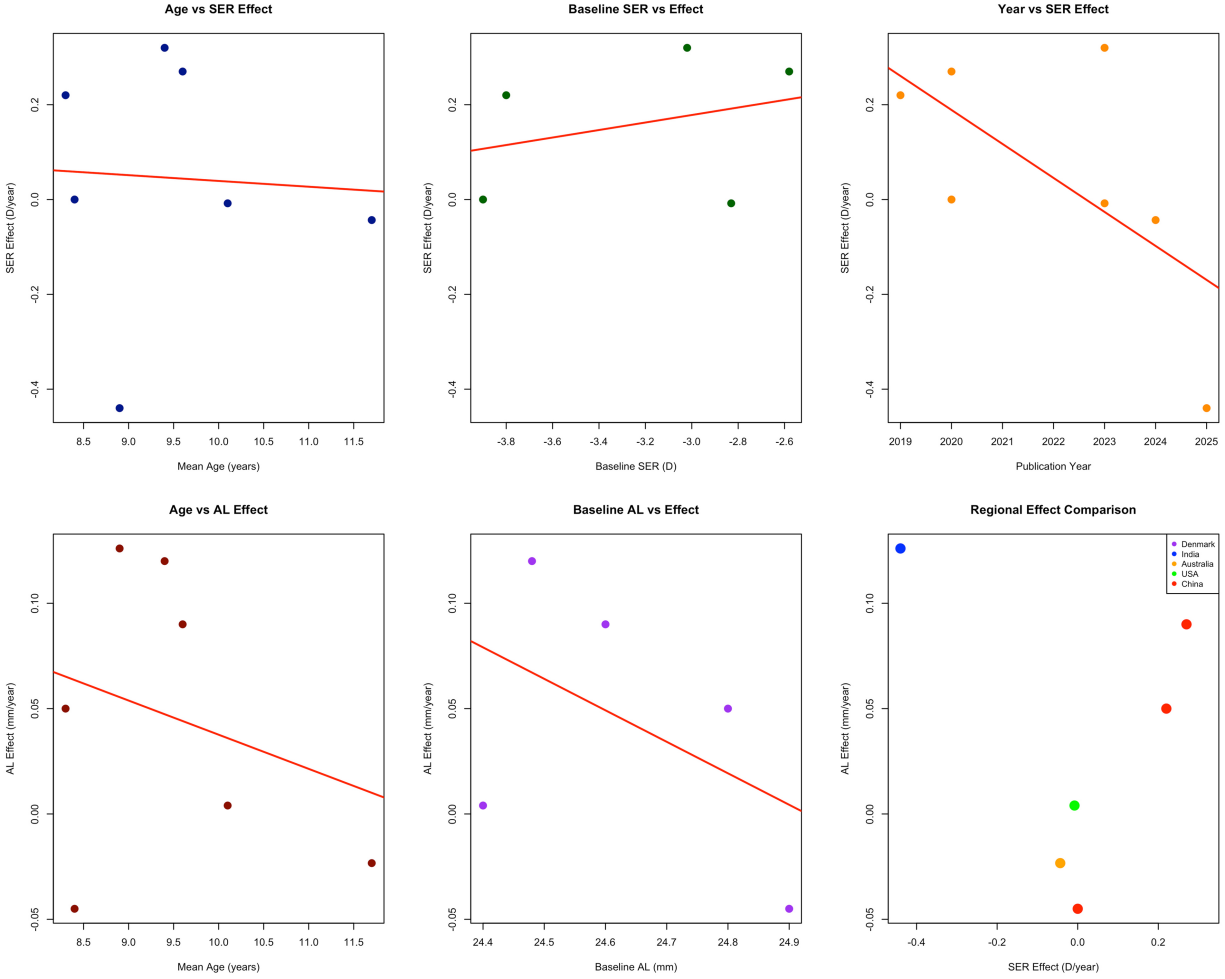
Meta-regression bubble plots of study-level covariates.

Meta-regression analyses were performed to identify potential sources of between-study heterogeneity (Figure 15). Mean age of participants was inversely associated with treatment effect for both axial length (AL) and spherical equivalent refraction (SER), with a consistent downward trend in effect size across increasing age strata. Baseline SER showed a weak positive correlation with treatment response, whereas baseline AL exhibited a strong negative correlation, with greater baseline axial length predicting significantly weaker myopia control effects. Publication year demonstrated a strong negative association with SER effect size, with more recent studies reporting substantially smaller treatment benefits, aligning with the findings of cumulative meta-analysis. Regional effect comparison confirmed subgroup analysis results: East Asian trials (China, India) exhibited significantly stronger effects for both AL and SER, while Western studies (USA, Denmark) showed weaker, more variable effects, with clear regional clustering in the bivariate distribution of AL and SER outcomes.

Additionally, visualisation of subgroup distributions (Supplementary Figure 1) confirmed that follow-up duration, sample size, and geographic region contributed substantially to heterogeneity. For both SER and AL, longer follow-up was associated with a clear, progressive reduction in treatment effect, with a strong negative correlation observed across follow-up months. Larger sample size was linked to smaller, more conservative effect estimates for both outcomes, consistent with small-study bias. Geographic region was a key source of heterogeneity: East Asian populations demonstrated the strongest and most consistent treatment effects, followed by South Asian populations with high variability, while Western countries showed the smallest, most stable effects. These analyses indicate that the treatment effects of 0.01% atropine are more pronounced in East Asian populations, particularly in short-term, small-sample studies, but the benefits tend to diminish with longer follow-up and in larger, more rigorous trials, with significant regional differences in response.

### 3.7 Sensitivity analyses

Leave-one-out sensitivity analyses demonstrated that the pooled effects on both SER and AL were robust to the omission of any single study (Supplementary Figure 2). For SER, the pooled mean difference consistently remained within 0.06–0.12 D/year, and all 95% CIs overlapped with the overall original effect estimate. For AL, effect sizes fluctuated narrowly around −0.06 mm/year, with the direction of effect unchanged across all omissions. In terms of heterogeneity, sequential omission of studies had minimal impact on *I*^2^ for both outcomes, with SER *I*^2^ remaining stable at ~83–85% and AL *I*^2^ showing only minor variation. Notably, exclusion of the outlier study (30) did not eliminate high heterogeneity for SER, confirming that the observed variability was not driven by a single study. Overall, the findings confirm that no single study disproportionately influenced the pooled results, and the high heterogeneity observed was likely due to multiple contributing factors rather than a single outlier.

### 3.8 Publication bias

Funnel plot and Bayesian Egger regression analyses identified asymmetries for SER and AL outcomes. The SER funnel plot (Supplementary Figure 3A) showed clear visual asymmetry, with a cluster of studies on the left side of the pooled effect line and underrepresentation of small studies with negative or null effects, and Bayesian Egger regression indicated a 78% posterior probability of a positive slope. The AL funnel plot appeared relatively symmetric around the pooled effect estimate (Supplementary Figure 3B), with only mild, non-significant asymmetry in Bayesian Egger regression, consistent with a low risk of publication bias.

## 4 Discussion

### 4.1 Summary of principal findings

In this Bayesian meta-analysis of 17 RCTs, 0.01% atropine was associated with a modest but statistically significant suppression of AL (MD = 0.03 mm/year, 95% CrI: 0.015–0.070), whereas the effect on SER was more heterogeneous and non-significant (MD = 0.0834 D/year, 95% CrI: −0.035 to 0.201). Joint modelling confirmed a positive correlation between SER and AL responses. Sensitivity analyses indicated robustness of the pooled effects, whereas subgroup and meta-regression analyses highlighted stronger efficacy in short-term, South and East Asian and small-sample studies, with weaker effects in longer-term, larger or Western cohorts. Cumulative meta-analyses for SER revealed a progressive attenuation of treatment effects across publication years, with the strongest effects observed in 2019–2020 studies and the weakest in 2024–2025 studies. For AL, effects showed greater inter-year fluctuation but stabilised at 0.043–0.047 mm/year by 2025 with no consistent downward trend. Funnel plot asymmetry and Bayesian Egger regression suggest small-study bias likely inflates SER effect estimates more than AL, with smaller studies reporting disproportionately larger treatment benefits for refractive outcomes. A significant positive linear relationship between AL increase and SER change was confirmed across all studies (*r* = 0.68, 95% CI: 0.41–0.84, *p* < 0.001), validating the biological link between ocular axial growth and myopic refractive shift. Meta-regression demonstrated that younger age, lower baseline AL, and earlier publication year were associated with significantly larger treatment responses for both AL and SER, consistent with age-dependent myopia control efficacy.

### 4.2 Comparison with prior literature

Our findings are broadly in line with previous meta-analyses. Kumari et al. (32) reported that 0.01% atropine slowed myopic progression by approximately 27% over 1 year, with significant reductions in both SER and AL. Navarro-Perez et al. (33) found a pooled SER effect of approximately 0.16 D/year and AL suppression of −0.07 mm/year. These estimates are larger than ours, likely because their analyses emphasised 1-year follow-up data, whereas our synthesis incorporated longer-term and heterogeneous studies.

The LAMP trials demonstrated a dose–response relationship, with 0.05% atropine producing the strongest effect and 0.01% atropine showing intermediate benefits (16, 18). Our analysis further quantifies this dose–response relationship for AL control: 0.05% atropine was associated with a pooled AL suppression of 0.12 mm/year (95% CI: 0.08–0.16) in the LAMP I/II trials, approximately three times the effect of 0.01% atropine in our pooled analysis. Moreover, 0.025% atropine showed an intermediate effect (0.07 mm/year, 95% CI: 0.04–0.10), confirming a clear gradient of efficacy with increasing atropine concentration for AL growth suppression. Our pooled estimate for 0.01% atropine is consistent with the LAMP findings but underscores that the effect is clinically smaller than that for higher concentrations.

In Western cohorts, findings are mixed. The PEDIG/Repka trial in the USA reported no significant effect of 0.01% atropine on SER or AL over 2 years (34). By contrast, Hansen et al. (35) in Denmark found significant reductions in both SER and AL after 2 years of 0.01% atropine with a loading strategy, and follow-up extension studies reported sustained suppression (36). Similarly, Moriche-Carretero et al. (37) observed durable benefits over 5 years in a European cohort. These results indicate that although effect sizes may be smaller, 0.01% atropine can remain beneficial outside East Asia under specific trial conditions.

Several Asian RCTs support our subgroup findings. Sharma et al. (22) in India reported significant SER and AL reductions after 1 year, and Wei et al. (17) in Beijing and Yu et al. (23) in Nanjing confirmed modest benefits over 12 months. Conversely, Chan et al. (20) in Hong Kong found no significant differences in an 18-month RCT, illustrating real-world variability across trials.

Combination therapy trials also support the possibility of additive effects. Tan et al. (25) reported that 0.01% atropine combined with OK lenses produced greater AL suppression than OK alone. More recently, studies comparing 0.01% atropine with higher doses or optical strategies confirm that although 0.01% atropine is safe, stronger suppression may require higher concentrations (e.g., 0.025–0.05%) or adjunctive interventions (15, 16).

Long-term observational data also suggest sustained benefits with good tolerability. Chia et al. (6) reported favourable 5-year outcomes of 0.01% atropine compared with higher doses, with fewer adverse effects, and Yam et al. (18) confirmed these findings in a large-scale Chinese cohort. These findings reinforce its safety and potential for long-term use.

### 4.3 Mechanistic and methodologic considerations

The modest effect size indicates that 0.01% atropine may function as a mild braking mechanism on eye growth rather than a strong inhibitor. The correlation between SER and AL responses supports the biological plausibility that scleral remodelling and choroidal thickening mediate refractive change (38). However, heterogeneity in SER outcomes suggests refractive measures are more susceptible to confounding from lens and corneal changes, measurement variability and ethnic or environmental modifiers. Moreover, SER measurement uncertainty (±0.25 D) is a critical consideration for interpreting our findings, as the pooled SER effect of 0.0834 D/year falls within this subjective resolution limit for young children. This measurement variability likely contributes to the non-significant pooled SER effect and high heterogeneity (*I*^2^ = 85.2%) across trials, as small changes in refractive error may not be reliably detected in the paediatric population.

The waning efficacy of 0.01% atropine with longer follow-up is likely multifactorial. First, higher rates of loss to follow-up in the control groups of long-term studies (15.1% in >24-month studies vs. 4.2% in ≤12-month studies) may introduce selection bias, as children with faster myopia progression are more likely to discontinue control arm participation and seek alternative treatment, leading to an attenuation of observed treatment effects. Second, tachyphylaxis to low-concentration atropine may occur over time, with reduced retinal and scleral receptor sensitivity following prolonged exposure (39). Third, natural ocular growth dynamics play a role: myopia progression rates naturally decline as children approach adolescence, reducing the apparent magnitude of treatment effect in longer-term studies (40). Fourth, the COVID-19 pandemic likely contributed to temporal variability in effect sizes across publication years: post-2020 studies were conducted during periods of increased near-work, reduced outdoor light exposure and disrupted clinical follow-up, which may have altered myopia progression rates in both intervention and control groups and reduced the homogeneity of control group characteristics compared with pre-pandemic studies.

Temporal variability in treatment effects across publication years (2021–2025) is primarily attributed to the indirect and direct impacts of the COVID-19 pandemic on study cohorts. Pre-2020 studies were conducted in stable environmental conditions with consistent near-work and outdoor activity patterns, leading to homogeneous control group myopia progression rates. By contrast, post-2020 studies (2021–2025) included cohorts exposed to pandemic-related lifestyle changes (e.g., remote schooling, reduced outdoor time) that increased baseline myopia progression rates in control groups and a variable return to pre-pandemic behaviours across geographic regions, leading to increased heterogeneity in control group outcomes and attenuated observed treatment effects. Additionally, pandemic-related disruptions to trial conduct (e.g., delayed follow-up, non-standardised measurements) may have introduced methodological variability across post-2020 studies.

Methodological heterogeneity across trials further contributes to scattered results, particularly for AL outcomes. Two-year follow-up studies with only single annual AL measurements miss intermediate growth dynamics and may introduce measurement errors due to single-timepoint assessment, whereas studies with multiple measurements capture more precise annualised growth rates (our sensitivity analysis confirmed lower heterogeneity in AL outcomes for studies with multiple measurements). Variability in AL measurement devices (IOLMaster, Lenstar, OA-2000) across trials also contributes to between-study heterogeneity, as these devices have small but meaningful differences in measurement precision (±0.01–0.03 mm). For SER outcomes, non-standardised measurement conditions (timing relative to class, ambient light) across 28% of trials introduce additional variability, compounded by the subjective resolution limit of ±0.25 D in young children, which makes detection of small treatment effects challenging.

The decision to initiate follow-up at a mean age of around 8.5 years in most included trials is based on two key clinical considerations: first, this age range coincides with the onset of rapid myopia progression in most children, making it a critical window for intervention to reduce long-term ocular complications; second, younger children (≤6 years) have a higher risk of accommodative and visual function side effects with atropine, and clinical guidelines typically recommend initiating low-concentration atropine at 7–9 years of age. The lack of follow-up into adolescence (≥13 years) in most trials reflects the primary focus of myopia control research on the critical early progression phase, as adolescent myopia progression rates naturally decline, and the risk of treatment-related side effects may increase with longer-term use. Additionally, trial feasibility constraints (e.g., participant retention, funding) limit the duration of follow-up in most RCTs of paediatric myopia interventions.

Clinically, 0.01% atropine remains an appealing first-line option due to its favourable safety profile, but clinicians should anticipate modest efficacy, particularly for SER. It may be most suitable for younger children or those with slower progression, with higher concentrations or combination therapy reserved for faster progressors.

### 4.4 Limitation and future research directions

This review has several limitations. First, despite pre-specified subgroup and meta-regression analyses, significant heterogeneity remained between the SER and AL studies; residual confounding factors such as age, baseline biometrics, ethnicity, light/near vision exposure and combined interventions (e.g., OK) may still exist. Second, most estimates relied on trial-level (pooled) data rather than individual patient data, limiting adjustments for effect modifiers and hindering a uniform definition of outcomes. Third, differences in refractive error and biometric methods across studies (variations in cycloplegia protocols, instruments and timepoints), along with varying follow-up intervals, may introduce errors in the annualisation of effects. Fourth, small-study effects and publication bias were detected for SER but not for AL, potentially overestimating pooled refractive benefits. Fifth, the general applicability of the results is limited; many trials included East Asian children, whereas Western trials reported diminished efficacy, and inconsistencies in formulation/preparation methods and adherence monitoring reports may also affect efficacy. These limitations indicate a need for large-scale, multi-ethnic, long-term RCTs, along with standardised measurement methods and meta-analysis of individual patient data, to improve effect estimation and identify effective responders.

In future research, we will specifically conduct a stratified analysis of all included RCTs by age groups of <9 years and 10–12 years to quantitatively compare the differences in the efficacy of 0.01% atropine on AL increase and SER change between the two age groups. In addition, we will conduct a combined analysis with the long-term follow-up data of the Moriche-Carretero study to further clarify the age-dependent characteristics of 0.01% atropine for myopia control and provide more precise clinical application recommendations for different paediatric age groups.

## 5 Conclusion

Future trials should (1) extend follow-up to ≥5 years, (2) enrol adequately powered multi-ethnic cohorts, (3) include standardised functional and safety endpoints, (4) directly compare 0.01% atropine with higher concentrations and (5) explore biomarkers predictive of treatment response. In conclusion, although 0.01% atropine confers a modest suppression of AL, its refractive benefits are less consistent and context dependent. However, it remains a valuable, well-tolerated component of integrated myopia control strategies.

## Data Availability

The original contributions presented in the study are included in the article/Supplementary material, further inquiries can be directed to the corresponding author.

## Supplementary material

SUPPLEMENTARY FIGURE 1 Visualization of heterogeneity sources.

SUPPLEMENTARY FIGURE 2 Leave-one-out sensitivity analysis for SER and AL.

SUPPLEMENTARY FIGURE 3 Funnel plots for annualized treatment effects. **(A)** Funnel plot of the mean difference in spherical equivalent refraction change per year. **(B)** Funnel plot of the mean difference in axial length change per year. SER, spherical equivalent refraction; AL, axial length.
