# Supplementary figures and images for: Efficacy of 0.01% atropine for myopia control in children: an artificial intelligence-assisted multivariate Bayesian meta-analysis
Source: Front Med (Lausanne). 2026 Jun 30;13:1752902. doi: 10.3389/fmed.2026.1752902 (PMC13364528; doi:10.3389/fmed.2026.1752902)

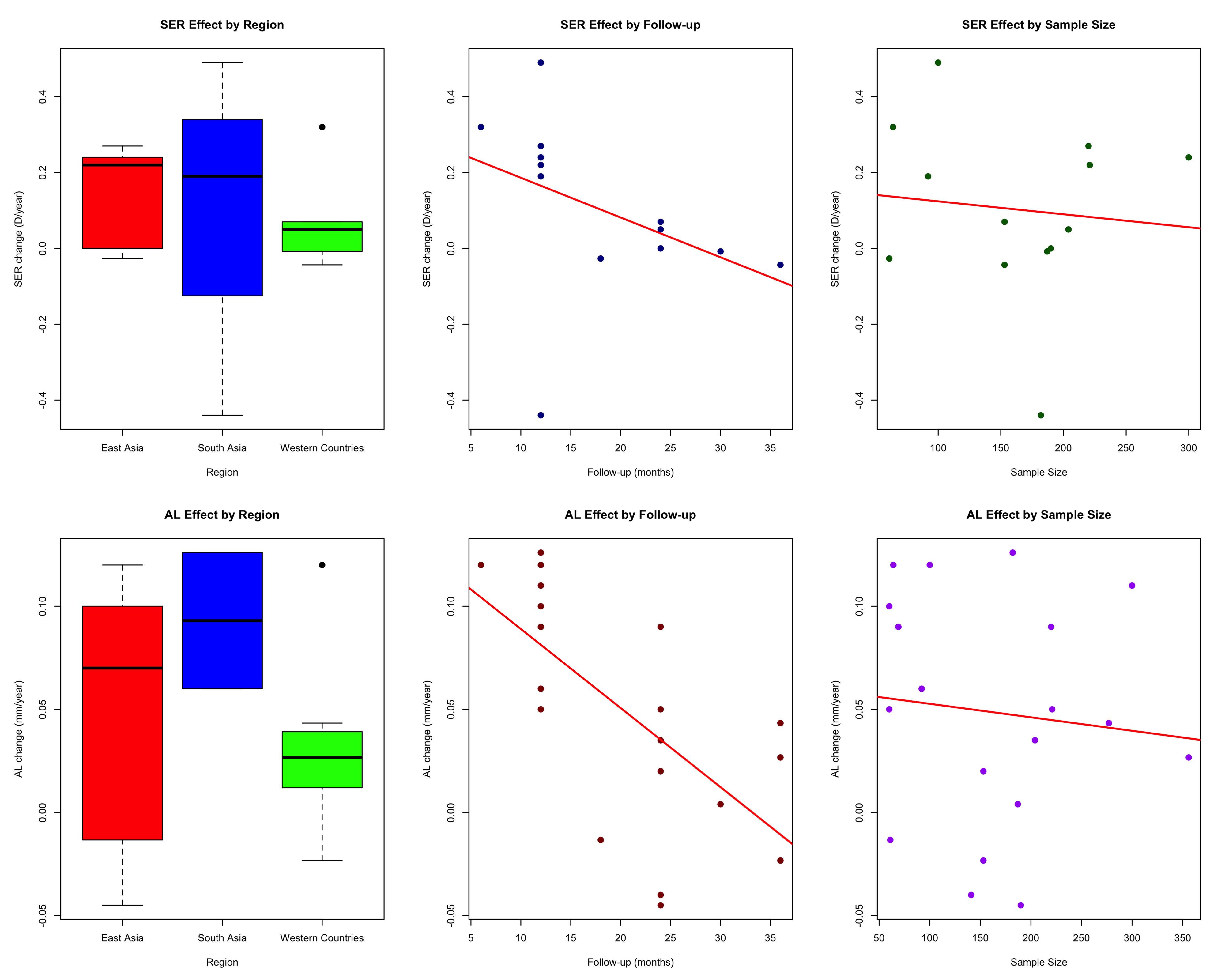

Supplement: SUPPLEMENTARY FIGURE 1 — Visualization of heterogeneity sources. [file Image_1.JPEG]

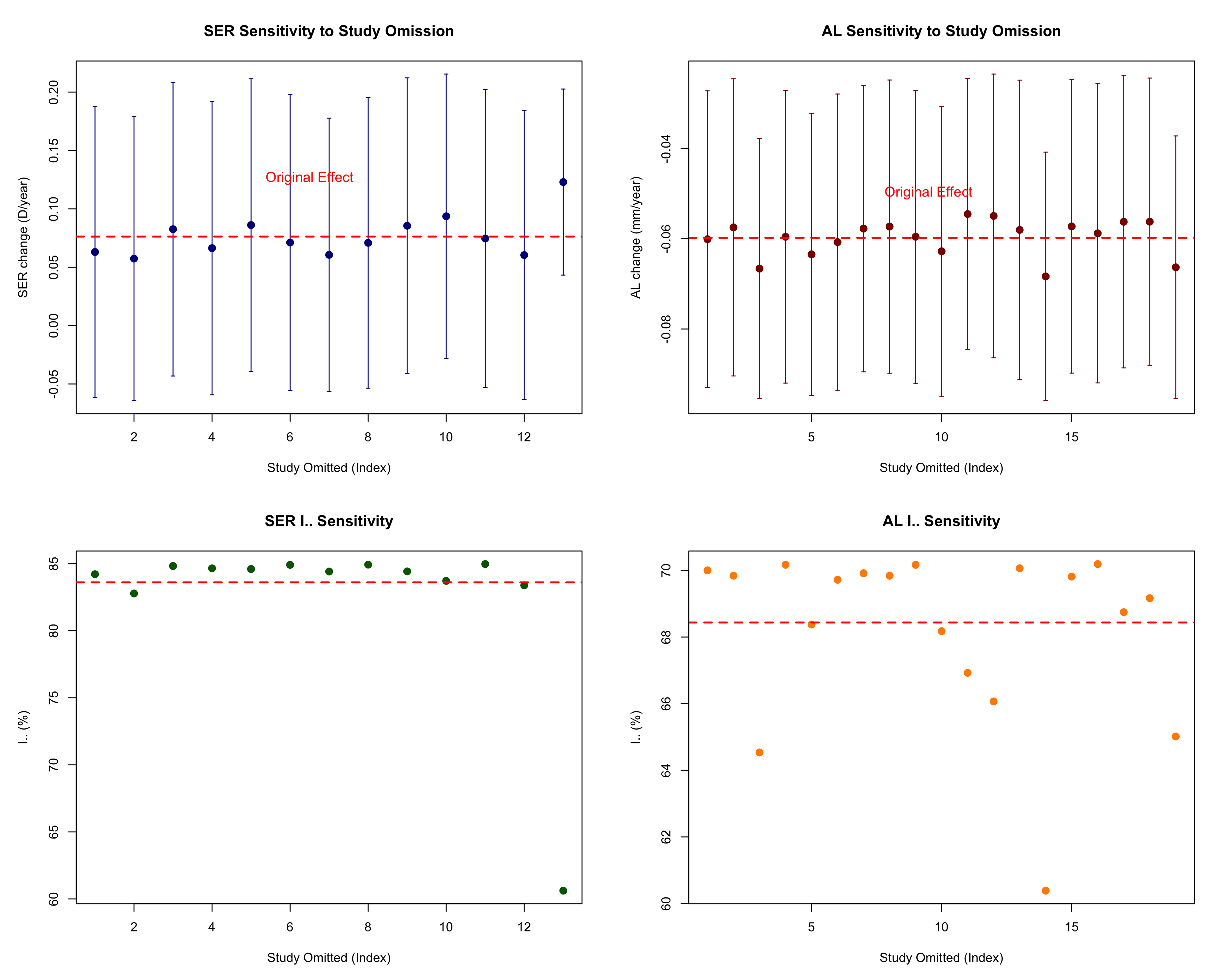

Supplement: SUPPLEMENTARY FIGURE 2 — Leave-one-out sensitivity analysis for SER and AL. [file Image_2.JPEG]

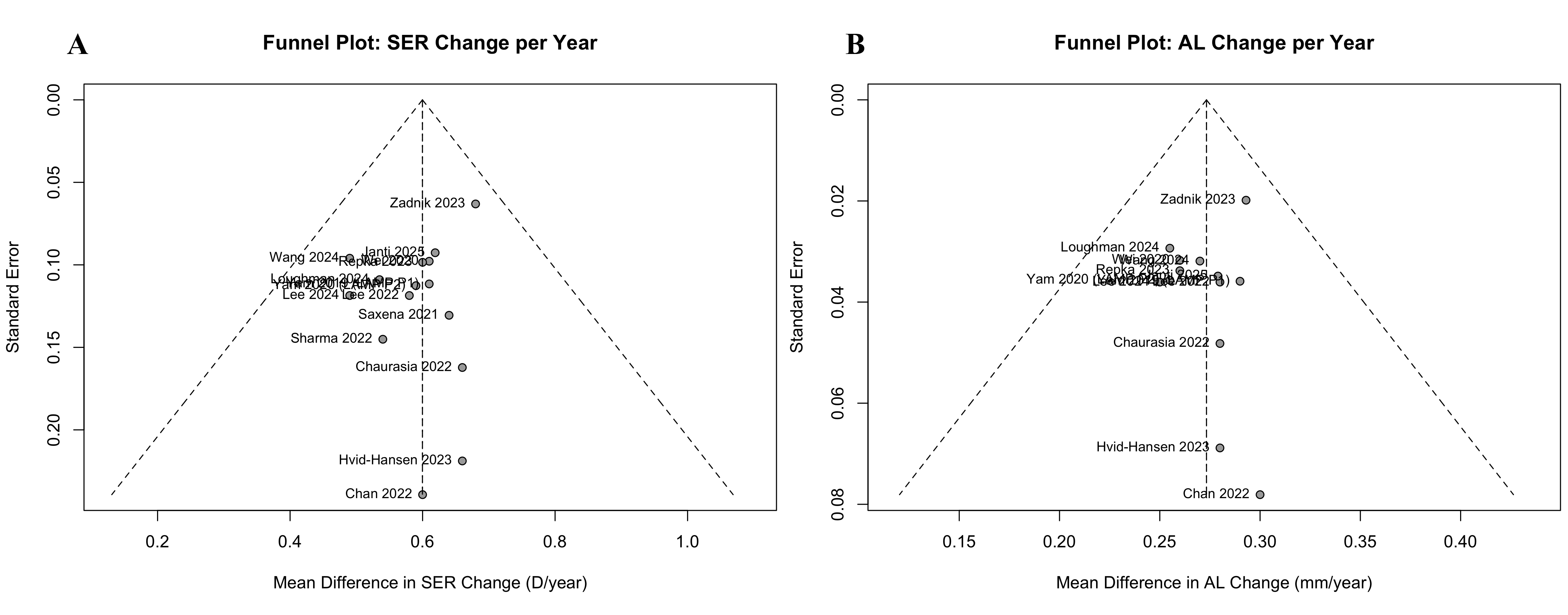

Supplement: SUPPLEMENTARY FIGURE 3 — Funnel plots for annualized treatment effects. (A) Funnel plot of the mean difference in spherical equivalent refraction change per year. (B) Funnel plot of the mean difference in axial length change per year. SER, spherical equivalent refraction; AL, axial length. [file Image_3.JPEG]
